# Pathological complete response achieved with FLOT chemotherapy in two patients with MSI-H esophagogastric junction and gastric adenocarcinoma

**DOI:** 10.1097/CAD.0000000000001652

**Published:** 2024-08-09

**Authors:** Federica Cosso, Daniele Lavacchi, Luca Messerini, Vittorio Briganti, Francesca Castiglione, Marco Brugia, Valentina Berti, Sara Fancelli, Fabio Cianchi, Agnese Vannini, Serena Pillozzi, Lorenzo Antonuzzo

**Affiliations:** a Clinical Oncology Unit, Careggi University Hospital; b Department of Experimental and Clinical Medicine, University of Florence; c Pathology Unit, Department of Experimental and Clinical Medicine, University of Florence; d Division of Nuclear Medicine, Careggi University Hospital; e Medical Oncology Unit, Careggi University Hospital; f Department of Biomedical, Experimental and Clinical Sciences ‘Mario Serio’, University of Florence; g Nuclear Medicine Unit, Azienda Ospedaliero-Universitaria Careggi; h Unit of Digestive Surgery, Careggi University Hospital, Florence, Italy

**Keywords:** chemotherapy, esophagogastric junction cancer, gastric cancer, high microsatellite instability, perioperative

## Abstract

Globally, more than 1 million new cases of gastric cancer were estimated in 2020, ranking fourth in cancer mortality. Currently although in resectable gastric cancer and esophagogastric junction (EGJ) adenocarcinoma a perioperative triplet chemotherapy regimen including a fluoropyrimidine, a platinum compound and docetaxel (FLOT) demonstrated a better overall survival, the survival rate is still very low, and a massive effort is still required to improve clinical prognosis. High microsatellite instability (MSI-H) status in gastric cancer is a favorable prognostic factor but poor data are available on its predictive role for perioperative FLOT chemotherapy in resectable gastric cancer. Here, we presented the case of two patients with advanced MSI-H gastric cancer/EGJ adenocarcinoma who had no residual tumor following neoadjuvant FLOT chemotherapy maintaining a complete response for more than 30 months, suggesting MSI-H status to be a positive prognostic marker also in patients treated with a taxane-containing triplet in this setting. We also discuss the future perspectives including the opportunity to achieve excellent clinical outcomes with immune checkpoint inhibitor (ICI)-based regimens.

## Introduction

Globally, more than 1 million new cases of gastric cancer were estimated in 2020, ranking fourth in cancer mortality [1].

While a gradual decline in the incidence of gastric cancer has been observed in Western Europe and North America, the incidence of esophagogastric junction (EGJ) adenocarcinoma seems to have moderately increased during recent decades [1,2].

Currently, perioperative therapy (pre- and postoperative) and radical gastrectomy is recommended in resectable gastric cancer and EGJ adenocarcinoma; a triplet chemotherapy regimen including a fluoropyrimidine, a platinum compound and docetaxel (FLOT) should be proposed to patients [2]. Unfortunately, the survival rate is still low, and a massive effort is still required to improve clinical prognosis [3].

The Cancer Genome Atlas TCGA identified a comprehensive set of genetic changes associated with gastric cancer as well as a guide to targeted agents. High microsatellite instability (MSI-H) gastric cancer is considered a distinct subtype and has higher mutation rates with unique DNA methylation patterns [4]. MSI-H has emerged as a favorable prognostic factor leading to prolonged survival while conflicting clinical data were reported on its potential negative predictive factor for neoadjuvant/adjuvant chemotherapy in resectable gastric cancer [5–7]. Since perioperative FLOT administration has become the new standard of care for locally advanced gastric cancer and EGJ adenocarcinoma more data on the use of this triplet in MSI-H patients are needed to verify its role as a predictor factor.

Here, we present the case of two patients with advanced MSI-H gastric cancer/EGJ adenocarcinoma treated with perioperative FLOT chemotherapy achieving pathological complete response (CR).

## Case presentation

### Case 1

**Fig. 1:**
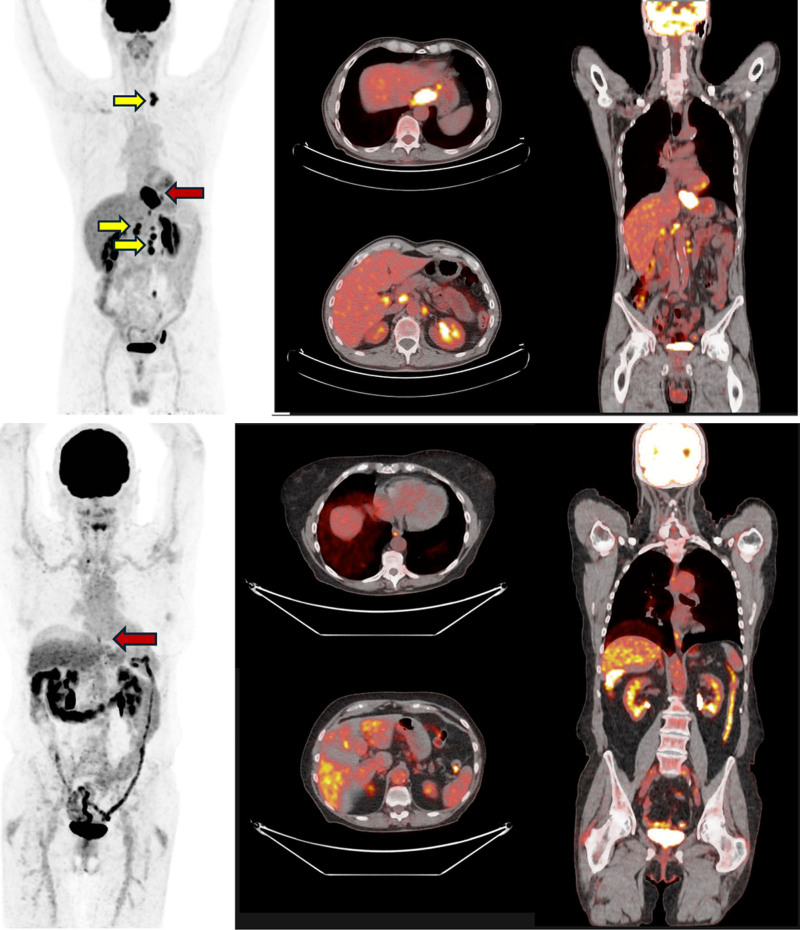
Baseline 18F-FDG PET/CT imaging for case 1 (upper) and case 2 (lower).

A 62-year-old male patient suffering from epigastralgia and weight loss was referred to our hospital. He had no other medical history. Esophagogastroduodenoscopy showed a stenosis in the distal esophagus (at 32 cm from the incisor teeth) due to a vegetative lesion. A histopathological examination of a biopsy specimen revealed a poorly differentiated (G3) adenocarcinoma. Biomarker analyses detected mismatch repair deficiency (dMMR) in immunohistochemistry, while HER2/neu resulted negative. Contrast-enhanced computed tomography (CT) scan did not reveal any invasion of the adjacent structures; however, six regional and distant swollen lymph nodes were detected and confirmed at the 18F-fluorodeoxyglucose PET (18F-FDG PET/CT) (Fig. 1): left retroclavicular region, left paraesophageal, lesser gastric curvature, hepatic hilum, paracaval, multiple left lumboaortic lymph nodes. We clinically diagnosed his tumor as oligometastatic disease cT2N2M1, cStage IV according to the Union for International Cancer Control TNM classification of malignant tumors, 8^th^ edition (UICC-TNM 8^th^). The biopsy of the left retroclavicular lymph node confirmed it as an oligometastatic adenocarcinoma site. The lab analysis, including complete blood counts, carcinoembryonic antigen, CA 19-9, was normal.

After multidisciplinary team (MDT) discussion, the patient received six cycles of FLOT (5-fluorouracil 2600 mg/mq reduced by 50% for DPYD mutation, oxaliplatin 85 mg/m^2^, docetaxel 50 mg/m^2^).

The subsequent CT scan revealed a partial response (PR) according to the Response Evaluation Criteria in Solid Tumors (RECIST 1.1) with a significant reduction in size of lymph nodes in the following regions: retroclavicular, lesser gastric curvature (the largest approximately 6 × 5 mm vs 13 × 9 mm), the hepatic hilum (the largest approximately 10 × 4 mm vs 18 × 12 mm), and in the left para-aortic area (the largest approximately 10 × 5 mm vs 18 × 13 mm).

Therefore, the patient underwent an Ivor Lewis esophagectomy + sampling of left para-aortic lymph nodes + interaortocaval + ijejunostomy, resulting in ypT0N0 stage, R0. The lymph node test results were as follows: bronchial and carenal (0/25), retroportal (0/6), para-aortic (0/10), aortocaval (0/4), periesophageal (0/8), paracardial (0/5) lymph nodes. No evidence of disease in surgical findings.

Thus, further four cycles of FLOT chemotherapy were performed to consolidate the CR, confirmed at the CT scan performed at the end of treatment.

The patient has been maintaining a CR for more than two and a half years.

### Case 2

A 73-year-old female patient with a history of hiatal hernia and persistent epigastralgia underwent an esophagogastroduodenoscopy identifying at the histopathological examination an adenocarcinoma of the angular incisure (or notch) associated with an ulcer (Forrest III). MSI-H was detected through PCR real time EasyPGX ready MSI (Diatech pharmacogenetics). HER2 expression by immunohistochemistry was negative, score 0, with a programmed death-ligand 1 (PD-L1) combined positive score (CPS) = 5.

The CT scan confirmed the 3 cm long-eccentric thickening starting from the distal esophagus to cardial region which did not reveal any invasion of the adjacent structures; however, four swollen regional lymph nodes were detected, negative at the 18F-FDG PET/CT (Fig. 1). We clinically diagnosed her tumor as cT3N0M0, cStage III according to the UICC-TNM 8^th^. The lab analysis, including complete blood counts, carcinoembryonic antigen, CA 19-9, was normal.

After the MDT discussion the patient received four cycles of FLOT chemotherapy. Docetaxel was omitted for cycle 4 due to an infusion reaction at the end of cycle 3.

The following CT scan showed a PR according to RECIST 1.1 criteria, allowing the Ivor Lewis subtotal esophagectomy plus D2 lymphadenectomy together with removal of lymph nodes in station 107, 108, 109R, 110 e 111 resulting in ypT0N0 stage, R0, AJCC 2017.

Accordingly, to complete the perioperative chemotherapy treatment, four cycles of 5-fluorouracil 2600 mg/mq, oxaliplatin 85 mg/m^2^ were administered, at a 20% reduced dose for a referred diarrhea G2 related to the treatment, according to the Common Terminology Criteria for Adverse Events (CTCAE) Version 5.0.

The patient has been maintaining a CR for more than 6 months.

## Discussion

In locally advanced resectable gastric cancer/EGJ the majority of patients may benefit from multimodal treatment. Thanks to the 5-year overall survival (OS) advantage (45 vs 36%) demonstrated in the FLOT4 study of the combination of perioperative triplet chemotherapy with FLOT over ECF/ECX (50 mg/m² epirubicin and 60 mg/m² cisplatin on day 1 plus either 200 mg/m² fluorouracil as continuous intravenous infusion or 1250 mg/m² capecitabine orally on days 1–21), the first scheme has become the standard treatment in Western Europe together with radical surgery [8].

Globally, MSI-H is observed in about 5–20% of patients with gastric cancer/EGJ mainly identified in early-stage cancers [9]. While older perioperative or adjuvant treatments failed in this population, the use of taxane-containing triplets is still poorly explored in literature as no systemic data is currently available.

Recently the two Italian, multicentric, observational RealFLOT and PROSECCO trials collected data from patients with resectable gastric cancer or GEJ adenocarcinoma treated with perioperative FLOT [6,10].

In RealFLOT MSS patients’ disease-free survival (DFS) was 17.4 months, whereas median DFS was not reached for MSI-H patients (95% CI 14.5 – NaN vs NaN, *P* = 0.270) [10].

In PROSECCO trial the median OS for MSS/pMMR pts was 34.84 months (95% CI 26.68–47.60), whereas was not reached for MSI-H/dMMR group (*P* = 0.0316). The median progression-free survival (PFS) for MSS/pMMR patients was 20.49 months (95% CI 17.83–25.2), whereas the median PFS was not reached in the MSI-H/dMMR group (*P* = 0.0325) [6].

Although limited in sample size and with a still immature follow-up, the data suggest MSI-H status to be a positive prognostic marker also in patients treated with a taxane-containing triplet in this setting.

We here presented two clinical cases of advanced MSI-H EGJ adenocarcinoma and gastric cancer who had no residual tumor following neoadjuvant FLOT chemotherapy evaluated at stage ypT0N0 maintaining a CR for more than 30 months, which could confirm previous clinical data. Finally, in our second case an overexpression of PD-L1 CPS was detected. In literature MSI has been recognized as a strong predictor of response to immune checkpoint inhibitor (ICI) treatment but the clinical value of PD-L1 expression is still under evaluation [11].

Currently, ICI efficacy in association with FLOT in resectable gastric cancer/EGJ adenocarcinoma is under investigation in several clinical trials: durvalumab (NCT04592913), pembrolizumab (NCT03221426), atezolizumab (DANTE trial, NCT03421288), and avelumab (NCT03399071) studies. The recent interim results of the phase I-II DANTE trial showed that adding atezolizumab to FLOT led to improved tumor downsizing and pCR rates (24 vs 15%; one-sided *P* = 0.032) Of note, regression rates further improved with higher PD-L1 expression (33 vs 12% in tumors with CPS ≥ 10) or in MSI-high tumors (63 vs 27%) [12].

Finally, the Italian multicenter, nonrandomized, single-arm, multicohort, open-label, phase II INFINITY study introduces a further potential practice changing strategy of nonsurgical management evaluating the combination of tremelimumab and durvalumab as a neoadjuvant (cohort 1) treatment or as a definitive treatment (cohort 2) in 18 patients with MSI-H/dMMR resectable cT2-4 any N G/EGJ adenocarcinoma [13]. Recently results showed that the pCR rate was 60% (9/15) and major-complete pathological response (<10% viable cells) was 80%. All patients with pCR had negative ctDNA status presurgery. pCR rate was 1/6 (17%) in T4 vs 8/9 (89%) in T2-3 tumors (*P* = 0.011), whereas no correlation was found with baseline N status. PD-L1 CPS was not associated with outcomes and TMB had a nonsignificant trend of correlation with pCR (median TMB 26 in non-pCR vs 40 in pCR group, *P* = 0.2) [13].

In conclusion, there are changing strategies for MSI-H EGJ/gastric cancer patients in perioperative setting. Our results confirm MSI-H as a robust prognostic marker and FLOT as a feasible option in certain circumstances. In the next future, it is likely that chemoimmunotherapy or immunotherapy alone may be the preferred treatment also in patients with resectable gastric cancer/EGJ adenocarcinoma [14].

## Acknowledgements

### Conflicts of interest

There are no conflicts of interest.
